# Case-Control Association Testing of Common Variants from Sequencing of DNA Pools

**DOI:** 10.1371/journal.pone.0065410

**Published:** 2013-06-07

**Authors:** Allan F. McRae, Melinda M. Richter, Penelope A. Lind

**Affiliations:** 1 University of Queensland Diamantina Institute, Brisbane, Australia; 2 Queensland Institute of Medical Research, Brisbane, Australia; University of Alabama at Birmingham, United States of America

## Abstract

While genome-wide association studies (GWAS) have been successful in identifying a large number of variants associated with disease, the challenge of locating the underlying causal loci remains. Sequencing of case and control DNA pools provides an inexpensive method for assessing all variation in a genomic region surrounding a significant GWAS result. However, individual variants need to be ranked in terms of the strength of their association to disease in order to prioritise follow-up by individual genotyping. A simple method for testing for case-control association in sequence data from DNA pools is presented that allows the partitioning of the variance in allele frequency estimates into components due to the sampling of chromosomes from the pool during sequencing, sampling individuals from the population and unequal contribution from individuals during pool construction. The utility of this method is demonstrated on a sequence from the alcohol dehydrogenase (*ADH*) gene cluster on a case-control sample for heavy alcohol consumption.

## Introduction

A large number of genetic associations with disease have been discovered in recent years [1]. However, it is likely that a large portion of these are purely associations, with the underlying causal variant(s) being in linkage disequilibrium with the associated variant. In order to identify the causal variant(s), we require firstly a complete catalogue of the genetic variation in the region, then identification of those variants that are associated with the disease, and finally functional studies to show which of these are causal.

While cost of sequencing individual samples is rapidly decreasing, it remains – and likely will remain for the immediate future – more cost efficient to identify all genetic variants through sequencing of DNA pools, followed by individual genotyping of the set of variants that are most associated with case/control status. While sequencing DNA pools presents challenges in accurately detecting rare variants with high sensitivity [2]–[4], this is relatively unimportant when following up an observed association with a common variant as it is unlikely that phenotypic associations with common variants are driven by single or multiple rare causal variants [5], [6].

Testing for association in DNA sequence from pools of cases and controls without correcting for the underlying sources of variation will not only result in a large inflation of the distribution of the test statistic relative to the null distribution [7], but also less obviously may result in the incorrect ranking of SNPs for follow-up. While methodology has been developed for case-control association analysis from DNA sequencing of pools, the majority have focused on the detection of association with rare variants [8], [9], while those for common variants provide complex and computationally intensive models [10], [11].

We describe a simple method that accounts for and readily quantifies the relative contribution of both the observable and non-observable sources of variation in the allele frequency estimates from DNA pools. The utility of this method in determining the relative impact of various aspects of study design on the variance of the association test-statistic is demonstrated using a case-control sample for heavy alcohol consumption and sequence from the alcohol dehydrogenase (*ADH*) gene cluster.

## Materials and Methods

We first describe a simple method for the analysis of case-control pool sequencing results, and then provide an example of its application.

### Testing for Association with DNA Sequence Data on Sample Pools

For each pool, 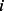, let 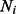 be the sequencing read depth at a SNP, 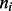 be the number of non-reference alleles seen at that SNP and 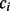 be number of chromosomes in the pool (i.e. twice the number of samples). Then the best estimate for the allele frequency of the SNP in the pool is 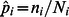. Then a simple test for the difference in allele frequency between two pools takes the form:
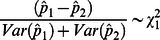



Three approaches to estimating 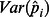 are investigated:

Variance #1: A naïve approach that assumes that the allele frequency estimate is equivalent to individual sequencing of the samples in the pool. In this case:
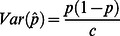



Variance #2: Correcting for all directly observable sources of variation in the allele frequency – the number of samples in the pool and the read depth in the pool. It can be shown that when accounting for these sources of variation, the variance of the allele frequency estimate is (see Appendix S1):
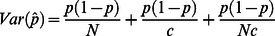



This splits the variation into three components; the variation caused by the random sampling of DNA molecules during the sequencing process, the variation caused through the sampling of individuals from the population in constructing the DNA pool and an interaction term that accounts for the non-independence of these two sampling processes. In practice the interaction term is much smaller than the two other terms and can be excluded from the calculation.

Variance #3: Correcting for both observable and non-observable sources of variation. Even with careful quantitation, there will be variation in the amount of DNA from each individual in the sample pool. This unequal contribution of DNA to the pool serves to inflate the portion of the variance in allele frequency estimates due to the sampling from the population. It can be shown that this inflation is constant with respect to allele frequency, and that the variance of the allele frequency estimate is:
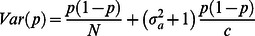
where 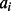 is the relative contribution of individual 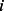 to the pool, scale such that 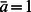 (Appendix S2). In an ideal setting, where the DNA contributions of all individuals to the pool are equal, 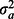 will equal zero and this equation reduces to Variance #2.

In practice, the relative contributions of an individual to the DNA pool is unknown. In order to estimate this variance from the data, an approach akin to genomic correction [12] can be used. Assuming the value of 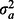 is equal for both the case and control pools, an iterative approach is taken in which 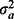 is adjusted until the median test statistic has the value expected from the 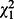 distribution. Note that this approach differs from standard genomic correction approaches, which multiply all test statistics by a constant factor to give the median test statistic its expected value, due to the presence of the variance term representing variation in sequencing read depth. While a direct estimate of this variance could be obtained through the completely independent construction of multiple pools from the same set of individuals, that is not considered here as it is inefficient and unlikely to be a common approach.

For the large-sample distribution of the test statistic for the difference in two proportions to apply, it is usually recommended that the samples size multiplied by the minimum of 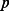 and 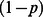 is greater than five. Due to the extra variance associated with sequencing read depth and pool construction, this threshold should be increased for the test statistic to satisfy its distributional requirements. In practical applications with pools involving several hundred people, filtering out all variants with less than 1% frequency is sufficient. Removing these variants also does not affect the follow-up of significant genetic associations results, as common variants are most likely to be underlying the observed association [5], [6].

### Case-control Testing of High Alcohol Consumption and the ADH Gene Cluster

As an example of the utility of this method, sequencing of a pooled case-control sample for alcohol consumption was performed. Consistent with previous work [13], [14], the heaviness of drinking measure for each individual was defined by a factor score with four components: lifetime maximum drinks, three heaviest period measures of frequency of heavy drinking, frequency of drinking to intoxication and average weekly consumption. From a population of 8,223 individuals in a family-based study, a sample of unrelated individuals consisting of 369 cases and 357 controls was selected, with cases defined as individuals having a factor score above 1.700 (range: 1.700 to 4.830; representing 8% of the population) and controls below −1.0135 (range: −1.0135 to −2.1267; the bottom 10.4%). Case and control pools were constructed and sequenced by Macrogen, Inc. Ethics approval for this study was provided by the Queensland Institute of Medical, Research Human Research Ethics Committee. Written and informed consent was provided by all study participants.

A 1 MB region on chromosome 4 (spanning from 99,653,607 to 100,610,500 – NCBI Build 36 / hg18) was sequenced, covering a number of alcohol dehydrogenase genes that have previously been implicated as being biologically relevant in phenotypic traits involved in the consumption of alcohol, including alcohol metabolism, subjective reactions to alcohol, excessive alcohol intake or alcohol dependence [15]–[18]. Cases were sequenced to an average read depth of 6311 (s.d. 2419) and controls 5744 (s.d. 2557) using an Illumina Genome Analyser IIx. Alignment of reads was performed using the software BWA [19] and variants were called using the pileup and varFilter options of the SAMtools [20]. A total of 1060 variants with a minor allele frequency greater than 1% in both cases and controls were called, consisting of 912 SNPs and 148 indels.

## Results

**Figure 1:**
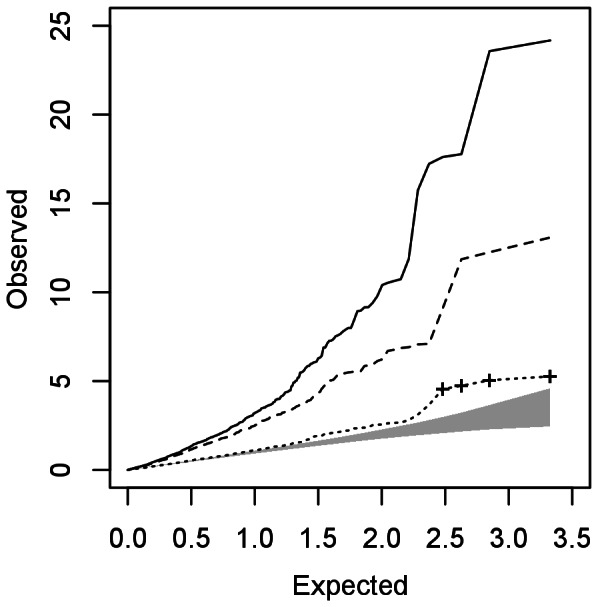
QQ-plot of -log_10_(p-value) from the test statistics using the three different estimates of the variance of the allele frequency in the sample pool: accounting for sample size in the pool (solid – variance #1), accounting for samples size and read depth (dashed – variance #2) and additionally accounting for variation in amounts of DNA in pool construction (dotted – variance #3). The 95% confidence interval for the expected distribution with no association and independent variants is shaded. The p-values for the four most significant variants are indicated with crosses.

The QQ-plot for the -log_10_(p-values) for the case-control test of heaviness of drinking is given in Figure 1. When calculating test statistics using variance estimates #1 and #2, there is a clear inflation of test-statistics as demonstrated by the genomic-inflation factor [12] being larger than 1 (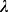 =  3.55 and 2.94 respectively). While the full correction using variance estimate #3 gives an inflation factor of 1 (by definition), there is still some deviation from the 95% confidence interval. This is not unexpected as the confidence interval is calculated assuming independent SNPs, which is clearly violated in data from DNA sequencing. Also, there is prior evidence of association for alcohol related phenotypes in this region that would indicate that some of this inflation may be caused by genuine association.

The four most significant results are all from indels that fall within a 60 KB region that spans ADH4 and its flanking regions. While individual genotype data is not available for this sample (and not all these variants are in the 1000 Genomes release from March 2012 [21], we can see that these variants are likely to be in strong LD using SNP data for this region from the CEU samples in the HapMap project [22]. Indeed, five of the next ten most significant variants are found in this region, presumably because of LD.

For the heavy alcohol consumption case-control pools, the value of 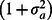 in variance estimate #3 is estimated to be 3.34. Using an average read-depth of 6000 and pool size of 320 individuals, this corresponds to the variation in sample contribution during pool construction being approximately 67% of the total variance in the allele frequency difference. This emphasises the importance of accurate pool construction for DNA sequencing case-control studies.

## Discussion

DNA pooling provides a cost effective mechanism for assessing common variation within a region in which a genetic association has been observed with the overall aim of identifying the causal variant(s). We have described a method for case-control association testing with data from DNA sequencing pools and have demonstrated its application to a cohort assessed for heaviness of alcohol consumption.

One important consequence of correcting for variation in the test-statistic caused by sequencing read-depth and pool construction is a potential change in the relative ordering of the largest test-statistics compared to a naïve comparison of allele frequency differences. This results in change to the priority of which variants should be followed up by individual genotyping. From the example dataset used here, only six of the ten most significant variants when using variance #3 are also in the list of top ten most significant variants when using variance estimate #1. In particular, two of the top ten variants when using variance estimate #1 are not in the list of the top 10% most significant variants when using variance #3, effectively demonstrating their initial association was a false positive.

Another point of interest is the relative contribution of variation introduced during the pool construction. In the example dataset used here, the pool construction accounted for the majority of variance in the test-statistic at all allele frequencies, with approximately 67% of the variation in allele frequency difference between the pools at the average read depth attributed to pooling variation. It is likely that this variance can be reduced though stringent quantitation during the construction of the equimolar DNA pools. This result is in contrast to DNA pooling on genotyping arrays where the variance introduced through DNA pool construction is small compared to that introduced from measuring the allele frequencies on the array [23]. This can be seen directly by noting that in the DNA sequencing of pools, the equivalent term to the array measurement variation is the variation from sampling DNA molecules during the sequencing process. Given any reasonable sequencing read-depth, this source of variation is likely to be the smallest component of the allele frequency variance.

While it remains to be seen how much the DNA pool construction variance can be reduced, its relatively large contribution to the variance indicates that approaches such as pooling of blood [24], where equal volumes will contain different amounts of DNA, may not be appropriate for sequencing studies. This can be seen by noting that the variance in allele frequency due to pool construction will be inflated by a further 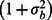, where 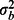 is the variance in the contribution to the DNA pool due to sampling from blood (when contributions are scaled to have an average of one), a typical value of which is around 0.25 [25]. In the example dataset used in this study, this would increase the contribution of pooling variation from 67% of the variance in allele frequencies to 73%. While this difference is not particularly large, the relative effect of the use of blood for pooling increases as the volume contributed by each individual becomes more even, meaning that any gains from accurate pipetting during pool construction will effectively be lost when pooling from blood.

The impact of sequencing error has not been included in the developed test-statistic. However, the effect of sequencing error is highest when investigating rare variants and the test-statistic provided here is only appropriate for common variants. Also, for an individual common variant, sequencing error using current technology is very low and will be orders of magnitude lower than the variance caused by finite read-depth, which is likely to be the smallest component of variance in the test-statistic for most studies. Not accounting for sequencing error variance will inflate the estimated variance due to unequal contributions of DNA to the pool to a very minor extent.

While the association test-statistic here only addresses the case of a single pool for the case and control sample, it does inform on the optimal approach for the use of more than one pool. Given the majority of the variation in the test-statistic is attributable to unequal contribution of DNA to the pool, it would be of most advantage to sequence independently generated DNA pools from the same individuals. However, accurate pool generation does require extensive investment in laboratory time and thus represents a trade-off given the use of DNA pools is primarily a cost reduction exercise.

The utility of using DNA sequencing on case-control pools was demonstrated using a sample selected on the basis of being at the extremes of a measure of heaviness of alcohol consumption. The most significant variants from the case-control analysis fall within a 60 KB region spanning the ADH4 gene. From publicly available individual level genotyping data for this region, it is likely that these variants are in high LD and therefore the associations will not be independent. While confirmation of significant association will require further follow-up with individual level genotyping and replication in an independent cohort, there have been a number of prior studies that have implicated this gene in susceptibility to alcohol dependence [16], [26]–[30]. Work is ongoing to validate these variants and their association in this sample using individual genotyping before performing the required replication that is needed in association studies.

One potential disadvantage of sequencing of DNA pools is the inability to remove individuals from the dataset retrospectively in order to control for potential population stratification. Even the individual sequencing of a particular genomic region is unlikely to provide enough information to adequately control for stratification. In the case of the test-statistic using variance #3, some of the effect of population stratification will be captured through the inflation of the estimated effect of the unequal contribution of individuals to the DNA pool. Not only does this reduce the power of the test, it potentially increases the rate of false positive results. Thus, care needs to be taken when selecting individuals for inclusion in the DNA pool. The issue of population stratification is readily avoided when using sequencing of DNA pools as a follow-up of a significant genome-wide association result, as the original SNP data from the GWAS provides information on population stratification in the sample.

In conclusion, we show that case-control pool sequencing can allow economical identification of poorly-tagged SNPs or other polymorphisms within a region identified by GWAS or because of its biological plausibility, and provide an example from our work on alcohol use and dependence.

## Supporting Information

Appendix S1 **Estimation of the variance in allele frequency difference between sequence from two DNA pools when accounting for pool sample size and sequencing read depth.**
(DOC)

Appendix S2 **Estimation of the effect of unequal contributions to the DNA pool on the variance of the estimate of the allele frequency in the pool.**
(DOC)
