# Supplementary material for: Case-Control Association Testing of Common Variants from Sequencing of DNA Pools
Source: PLoS One. 2013 Jun 7;8(6):e65410. doi: 10.1371/journal.pone.0065410 (PMC3676437; doi:10.1371/journal.pone.0065410)
Supplement: Appendix S1 — Estimation of the variance in allele frequency difference between sequence from two DNA pools when accounting for pool sample size and sequencing read depth. (DOC) [file pone.0065410.s001.doc]

**Appendix S1:** Estimation of the variance in allele frequency difference between sequence from two DNA pools when accounting for pool sample size and sequencing read depth.

Let $N$ be the sequencing read-depth of a pool, $n$ be the number of non-reference alleles seen in the sequencing and $c$ be the number of chromosomes sampled in pool construction (i.e. twice the number of individuals in the pool). Also, let $f$ be the unobserved frequency of the non-reference allele in the pool and $p$ be the unobserved population non-reference allele frequency. We want to estimate $Var\left(p\right)$, the variance of the estimated population allele frequency.

From the standard properties of the binomial sampling distribution, we know:

$$E\left(f\right)=p$$

and

$$E\left(f^{2}\right)=\frac{p}{c}+\frac{c-1}{c}p^{2}$$

Similarly,

and

Substituting the expected values for $f$ and $f^{2}$, we obtain:

Thus,

Rearranging this gives

$$Var\left(\hat{p}\right)()=\frac{p\left(1-p\right)}{N}+\frac{p\left(1-p\right)}{c}-\frac{p\left(1-p\right)}{Nc}$$

which splits the variance of $\hat{p}$ into three components representing (from left to right) the sampling of chromosomes from the DNA pool while sequencing, the sampling of chromosomes from the population while constructing the pool and an interaction term reflected the non-independence of these two sampling stages.
