# Supplementary material for: Case-Control Association Testing of Common Variants from Sequencing of DNA Pools
Source: PLoS One. 2013 Jun 7;8(6):e65410. doi: 10.1371/journal.pone.0065410 (PMC3676437; doi:10.1371/journal.pone.0065410)
Supplement: Appendix S2 — Estimation of the effect of unequal contributions to the DNA pool on the variance of the estimate of the allele frequency in the pool. (DOC) [file pone.0065410.s002.doc]

**Appendix S2:** Estimation of the effect of unequal contributions to the DNA pool on the variance of the estimate of the allele frequency in the pool.

Let $c$ be the number of chromosomes sampled in pool construction (i.e. twice the number of individuals in the pool), $p$be the unobserved population non-reference allele frequency and $a_{i}$ be the relative contribution of chromosome $i$ to the pool, scaled such that $\sum\left(a_{i}\right)=c$ and $g_{i}$ be the genotype of chromosome $i$. Then frequency of the non-reference allele in the pool is estimated as

$$\hat{p}=\frac{\sum_{i=1}^{c} a_{i}g_{i}}{c}$$

and the variance of this estimate is

$$Var\left(\hat{p}\right)()=Var\left(\frac{\sum_{i=1}^{c} a_{i}g_{i}}{c}\right)=\frac{\sum_{i=1}^{c} a_{i}^{2}}{c^{2}}Var\left(g_{i}\right)=\frac{\sum_{i=1}^{c} a_{i}^{2}}{c}\frac{p\left(1-p\right)}{c}$$

Comparing this variance to the equivalent term in in the variance provided in Appendix 1, it is seen that unequal contributions to the DNA pool inflates the variance attributable to sampling from the population by a factor of $\sum\left(a_{i}^{2}\right)/c$. Note that

$$Var\left(a_{i}\right)=E\left(a_{i}^{2}\right)-E\left(a_{i}\right)^{2}=\frac{\sum\left(a_{i}^{2}\right)}{c}-\left(\frac{\sum a_{i}}{c}\right)^{2}=\frac{\sum\left(a_{i}^{2}\right)}{c}-1$$

As variances are greater than or equal to zero by definition, we see that the scaling factor is always greater than or equal to one and reaches its minimum when all individuals contribute equally to the pool.
